# Dissecting beta-state changes during timed movement preparation in Parkinson’s disease

**DOI:** 10.1016/j.pneurobio.2019.101731

**Published:** 2020-01

**Authors:** Simone G. Heideman, Andrew J. Quinn, Mark W. Woolrich, Freek van Ede, Anna C. Nobre

**Affiliations:** a Oxford Centre for Human Brain Activity, Wellcome Centre for Integrative Neuroimaging, Department of Psychiatry, University of Oxford, Oxford, United Kingdom; b Department of Experimental Psychology, University of Oxford, Oxford, United Kingdom

**Keywords:** Beta oscillations, Burst-events, Parkinson’s disease, Movement, Timing

## Abstract

An emerging perspective describes beta-band (15−28 Hz) activity as consisting of short-lived high-amplitude events that only appear sustained in conventional measures of trial-average power. This has important implications for characterising abnormalities observed in beta-band activity in disorders like Parkinson’s disease. Measuring parameters associated with beta-event dynamics may yield more sensitive measures, provide more selective diagnostic neural markers, and provide greater mechanistic insight into the breakdown of brain dynamics in this disease. Here, we used magnetoencephalography in eighteen Parkinson’s disease participants off dopaminergic medication and eighteen healthy control participants to investigate beta-event dynamics during timed movement preparation. We used the Hidden Markov Model to classify event dynamics in a data-driven manner and derived three parameters of beta events: (1) beta-state amplitude, (2) beta-state lifetime, and (3) beta-state interval time. Of these, changes in beta-state interval time explained the overall decreases in beta power during timed movement preparation and uniquely captured the impairment in such preparation in patients with Parkinson’s disease. Thus, the increased granularity of the Hidden Markov Model analysis (compared with conventional analysis of power) provides increased sensitivity and suggests a possible reason for impairments of timed movement preparation in Parkinson’s disease.

## Highlights

• Amplitude changes of 15−28 Hz beta activity can have distinct single-trial causes.
• Use of HMM to disentangle constituents of trial-average changes in beta amplitude.
• Dissect beta state changes during timed movement preparation in PD and controls.
• Reduced temporal preparation effects on behaviour and on event interval times in PD.
• Event-based characterisation affords greater granularity and higher sensitivity.

## Graphical abstract

**Figure:**
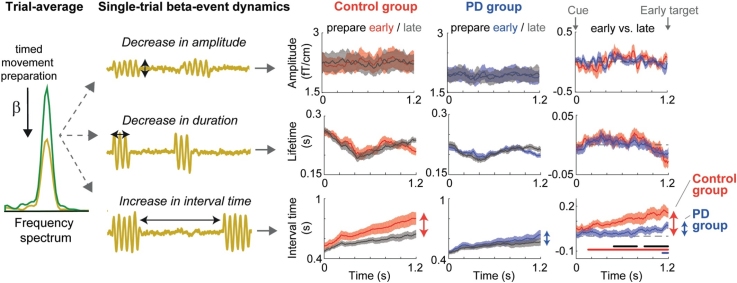

## 1 Introduction

Beta-band activity (15–28 Hz) is one of the most prevalent frequency-specific patterns of activity across both cortical and subcortical areas in the human brain. Aberrant beta-band activity has been implicated in multiple neurological disorders, such as Parkinson’s disease (Hammond et al., 2007; Little and Brown, 2014); Alzheimer’s disease (Koelewijn et al., 2017) and Amyotrophic Lateral Sclerosis (Proudfoot et al., 2017, 2018). Measures of beta-band activity may thus provide sensitive markers of abnormality in brain circuits, but more mechanistic and sensitive measures are likely to be required to aid selective diagnosis or prognosis.

**Fig. 1:**
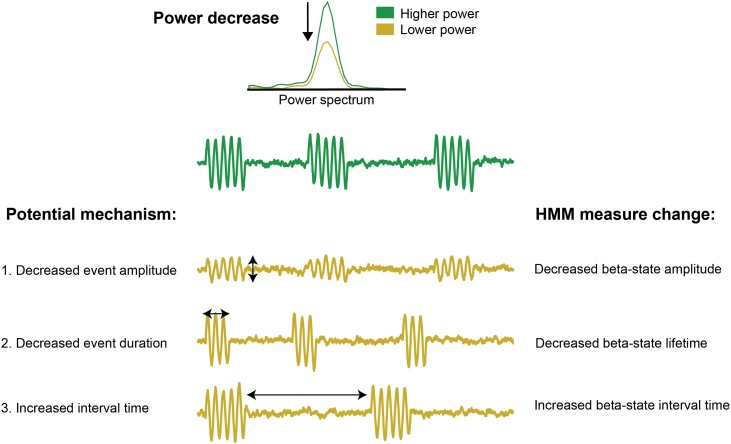
**Potential mechanisms of (beta) power decrease.** There are at least three mechanisms that could lead to a decrease in power (as depicted at the top) as observed in conventional analysis of trial-averaged power. The green time course at the top is a schematic of beta events during the higher power (initial) situation, while the yellow time courses below it show changes in event characteristics that could lead to a power decrease. The three potential mechanisms are: 1) a decreased event amplitude (mechanism 1 in the schematic); 2) a decreased event duration (mechanism 2); 3) an increased interval time between consecutive events, which corresponds to a decrease in the rate of occurrence of events (mechanism 3). The corresponding change in our HMM analysis is respectively, a decreased beta-state amplitude, a decreased beta-state lifetime, and an increased beta-state interval time.

In most studies to date, the description of beta-band changes is limited to variations in overall beta power during rest or task responses. Recently, it has been suggested that frequency-specific patterns of brain activity consist of short-lived, isolated, high-amplitude events (Feingold et al., 2015; Jones, 2016; Lundqvist et al., 2016; Sherman et al., 2016; Vidaurre et al., 2016; Shin et al., 2017; Tinkhauser et al., 2017a, 2017b; 2018; van Ede et al., 2018; Little et al., 2019), which only appear sustained when averaged across trials. By adopting this perspective, it is possible to consider multiple parameters that may influence overall beta power, like changes in event amplitude, in event duration, or in the time between subsequent events (see Fig. 1). To investigate high-amplitude beta events we need methods that can distinguish such events from surrounding ongoing (lower amplitude) activity with high temporal resolution, on a single-trial level.

In the current study, we investigated changes in beta activity between individuals with Parkinson’s disease and control participants during timed movement preparation using magnetoencephalography (MEG). We used a novel Hidden Markov Model (HMM)-based approach (as in Baker et al., 2014; Vidaurre et al., 2016) to compare various parameters (amplitude, lifetime, and interval time; see Fig. 1) to characterise changes in beta with greater sensitivity and mechanistic granularity. In contrast to amplitude thresholds that provide a simple and an intuitive method for beta-event characterisation, the HMM has one important practical and one important theoretical advantage. Practically, it does not require the specification of an a priori amplitude threshold. Adding to this, an important theoretical advantage of the HMM is that the dynamics have some temporal regularisation. In other words, a brief dip in amplitude during a period of high amplitude is not likely to lead the HMM to change to the “off” state. In contrast, when using a simple threshold, small noisy changes in amplitude close to the chosen threshold can lead to a period of high amplitude being split into many small events.

Evaluation of single-trial changes in the beta state using the HMM allowed us to distinguish between three distinct scenarios (Fig. 1). First of all, beta power could decrease because of a decrease in beta-event amplitude, evident as a decrease in the amplitude of the HMM beta state. Second, the duration of events could decrease, which would be captured by a decrease in the lifetime of the HMM beta state. Finally, the interval between subsequent events could increase, which would appear as an increase in HMM beta-state interval time.

We found that decreases in overall beta power during movement preparation were exclusively associated to the interval-time variable. By considering the single-trial changes in this parameter, were we able to reveal robust differences between the Parkinson’s disease group and the matched healthy control group. These findings show that the HMM is a powerful method that can differentiate between different factors that constitute beta-power changes and thereby increase our mechanistic understanding of the breakdown of such changes in disorders such as Parkinson’s disease.

## 2 Material and methods

### 2.1 Participants

The study protocol was approved by the Oxfordshire Research Ethics Committee as part of the National Research Ethics Service (Reference number 12/SC/0650). Nineteen individuals with idiopathic Parkinson’s disease (PD) and twenty-one age- and education-matched healthy control participants completed the study following a screening procedure. The screening procedure established eligibility for both MEG and MRI, though the current report focuses exclusively on the MEG experiment. Informed consent was obtained according to the Declaration of Helsinki; participants were reimbursed for their time and travel expenses. PD participants were recruited via the Dementias and Neurodegeneration Specialty (https://dendron.org.uk). The following set of inclusion criteria were adopted: (a) having a diagnosis within 5 years of the participation date, (b) being able to understand instructions in written and spoken English, (c) being above the age of 50, and (d) being able to tolerate coming off PD medication. Healthy older adults were recruited from the Oxford Dementia and Ageing Research database (https://www.oxdare.ox.ac.uk/) and were selected based on being similar in age and education to the PD participants.

All but two patients and two healthy control participants were right handed, and all participants had normal or corrected-to-normal vision. Data from three control participants and one PD participant were excluded for the following reasons. Data from two control participants were excluded because of technical problems during MEG data acquisition, resulting in the loss of trigger information. Data from one control participant and one PD participant were excluded because of their inability to comply with task instructions. The resulting analysis included data from eighteen PD participants (10 males, 8 females; aged 68.5 ± 6.5 SD) and eighteen matched control participants (9 males, 9 females; aged 67.3 ± 4.8 SD). PD participants were asked to withdraw from their dopaminergic medication starting from 19:00 h the night before the experiment, and to refrain from taking their medication in the morning.

At the start of the MEG session, we evaluated participants’ cognitive abilities (Montreal Cognitive Assessment version 7.1, MoCA; Nasreddine et al., 2005). In addition, participants were examined by a trained clinician using the Unified Parkinson’s Disease Rating Scale (UPDRS; Goetz et al., 2008). As part of this rating, disease severity was evaluated using the Hoehn and Yahr (1967; H & Y) scale. A summary of demographics, MoCA, UPDRS and H & Y scores for both groups is shown in Table 1. Individual UPDRS scores, H & Y scores, most affected side, years since diagnosis, and Levodopa-equivalent daily dose are shown in Supplementary Table 1.

**Table 1:** Summary of demographics and clinical scores for PD group and control group.

	PD Group (N = 18, 8 F)		Control group (N = 18, 9 F)		
	Mean ± SEM	Range	Mean ± SEM	Range	p
Age	68.5 ± 1.5	54 – 79	67.3 ± 1.1	60 - 76	n.s.
Education	13.8 ± 0.8	10 – 23	15.1 ± 0.9	10 - 20	n.s.
MoCA	26 ± 0.8	18 – 29	28.3 ± 0.3	26 - 30	.012
UPDRS-III	31.5 ± 3.1	11 – 51	1.3 ± 0.4	0 - 4	< .001
H & Y	1.75 ± 0.1	1 – 3	0	0	< .001

Age: age in years; Education: education in years; MoCA: Montreal Cognitive Assessment; UPDRS-III: Unified Parkinson’s Disease Rating Scale section III; H & Y: Hoehn and Yahr scale; p: probability of difference between PD and healthy control participants, Mann–Whitney non-parametric test (n.s.: non-significant).

### 2.2 Experimental setup

All participants were tested with MEG at the Oxford Centre for Human Brain Activity using an Elekta NeuroMag (306 channel) MEG system. A magnetic Polhemus FastTrak 3D system (Vermont, United States) was used for head localisation. Relative positions of three anatomical landmarks (nasion, left and right auricular points) were measured in addition to relative positions of four head-position indicator coils.

MEG data were recorded in six separate blocks of 5−6 min each. In between blocks, participants had a small break, during which they remained seated in the MEG chair. During acquisition, an analogue bandpass filter between 0.03 and 300 Hz was applied and data were digitised at a sampling rate of 1000 Hz. ECG and horizontal and vertical EOG were recorded in addition to eye tracking data that were recorded with a video-based eye tracker (EyeLink 1000, SR Research, Ontario, Canada) with a sampling frequency of 1000 Hz. A bimanual fibre-optic response device was used to collect manual responses.

Stimuli were presented using MATLAB (The MathWorks, Inc., Natick, MA) and Psychtoolbox v.3.0 for MATLAB (Kleiner et al., 2007). The stimuli were back-projected (Panasonic PT D7700E, Panasonic, Osaka Japan) on a 58 × 46 cm screen placed 120 cm in front of the participant, with a spatial resolution of 1280 × 1024 and a refresh rate of 60 Hz.

### 2.3 Experimental procedure and stimuli

**Fig. 2:**
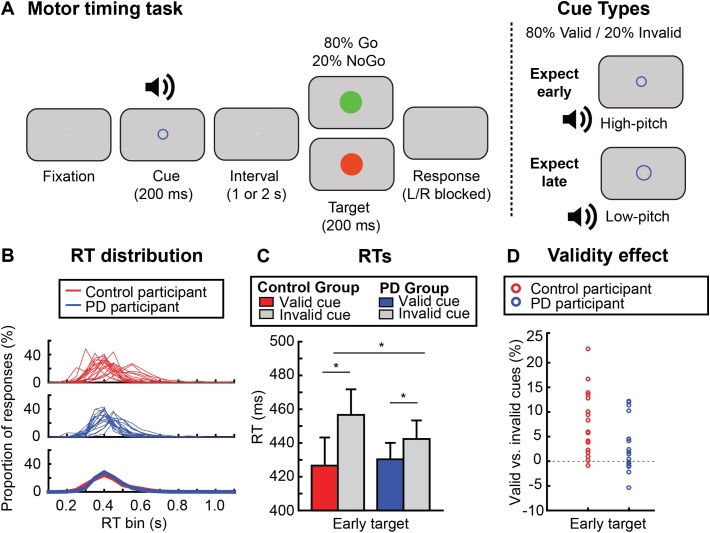
**Experimental task and behavioural results.** (A) A combined auditory-visual cue, consisting of a blue circle and an auditory beep, predicted when (with 80 % validity) a subsequent Go/NoGo target would occur. The small circle/high-pitched beep combination indicated that the target was likely to occur early (1-s inter-stimulus-interval), while a larger circle/lower-pitched beep indicated that the target was likely to occur late (2-s inter-stimulus interval). Green Go targets were presented in 80 % of trials; red NoGo targets were presented in 20 % of trials. Participants were instructed to respond as quickly as possible whenever a green target appeared, but to withhold responding to the red target. Note that stimuli are shown here larger than true size and on a grey background for display purposes only; in the actual experiment stimuli were presented on a black background instead. (B) Reaction time (RT) distributions for each participant displayed as the proportion of responses within 50-ms bins between 0 and 1.1 s. Control participants are shown in red and PD participants are shown in blue. Thick lines in the bottom plot indicate group averages and reveal highly comparable distributions. (C) RT results shown separately for healthy control participants and PD participants, for early targets following valid and invalid temporal cues (i.e. cues that predicted an early vs. a late target). Note that we focused on this early interval (in both the behavioural and the MEG data) given that temporal orienting effects are known to be largely restricted to this interval (see e.g. Nobre and van Ede, 2018). Error bars show ±1 standard error of the mean(SEM). Asterisk indicate statistically significant effects. (D) The size of the temporal validity effect (relative difference in RT following a valid vs. invalid cue for targets presented at the short interval) for each individual, shown separately for both groups.

Participants performed a cued Go/NoGo task, which is shown in Fig. 2A. A combined auditory-visual cue predicted with 80 % validity when (early: 1-s cue-target inter-stimulus interval; late: 2-s inter-stimulus interval) a subsequent Go/NoGo target would appear. A smaller circle (diameter: 0.57° of visual angle) combined with a high-pitched beep (880 Hz) predicted an early Go/NoGo target (diameter: 1.53° of visual angle), and a larger circle (diameter: 1.34° of visual angle) combined with a low-pitched beep (440 Hz) predicted a late Go/NoGo target. Participants had to respond as quickly as possible by pressing a button on a button box whenever a green target appeared (the Go target; present in 80 % of trials) and to withhold responding whenever a red target was presented instead (the NoGo target; present in 20 % of trials). A central fixation dot (diameter: 0.01° of visual angle) was present throughout the trial. Visual cues and targets were presented for 200 ms each, and auditory beeps were presented for 100 ms. After presentation of the Go target participants had 2 s to respond. After the response there was a 3–5 second interval before the start of the next trial. Note that for clarity in Fig. 2A all stimuli are shown larger than true size and on a grey background. In the actual experiment stimuli were presented as described above, and on a black background.

Six blocks of fifty trials each were presented in total. Responses were made with the left or right index finger. The response hand alternated between blocks (but always remained the same throughout the block) and the side that participants started with was counterbalanced across participants. Preceding each block, the hand to be used was indicated on the screen. After each block there was a break, during which the MEG data were saved. Participants were instructed on the task preceding the MEG recording. They were told the predictive nature of the cues and were encouraged to use this information to guide their behaviour. Participants performed 30 practice trials whilst seated in the MEG chair before starting the main experiment.

### 2.4 Behavioural analysis

The behavioural data were analysed using MATLAB, and statistics were performed in SPSS version 22 (IBM Corp. Armonk, NY). The behavioural data were analysed with respect to reaction times (RTs) and percentage correct (PC).

For the RT analysis (Go trials only), trials with an RT smaller or larger than a participant’s mean RT ± 3 times the standard deviation were excluded. On average 2.7 ± 0.2 trials were excluded this way. Trials with anticipatory responses were removed from the analysis (mean ± SD control group: 0.48 ± 0.55 % of trials; PD group: 1.30 ± 2.40 % of trials). The analysis of PC was performed separately for Go and NoGo trials. Correct trials in the analysis of Go trials were trials where a response was made (on average 98.1 ± 0.7 % of trials), while correct trials in the analysis of NoGo trials were trials in which participants did not respond (on average 84.0 ± 2.3 % of trials). For all reported behavioural analyses, we performed a repeated-measures ANOVA with the within-subject factors “Cue Validity” (valid or invalid) and the between-subjects factor “Group” (control or PD participants). Post-hoc pairwise t-tests were performed only if the interaction between Cue Validity and Group was significant.

The relationship between clinical symptoms in the PD group and the behavioural data was evaluated by calculating Pearson correlation coefficients between UPDRS-III scores and the behavioural results (see Supplementary Materials). This analysis was performed both for average RT scores (across all conditions) and the temporal validity effect at the short interval (relative difference in RTs following valid vs. invalid temporal cues).

### 2.5 MEG analysis

#### 2.5.1 Preprocessing and artefact rejection

MEG data preprocessing was performed using Matlab and OSL version 2.0 (https://ohba-analysis.github.io/osl-docs/). First, channels containing excessive noise were identified automatically using MaxFilter version 2.2.15 software (Neuromag). Subsequently, MaxFilter’s spatiotemporal signal space separation (Taulu et al., 2004; Taulu and Simola, 2006) and movement compensation were applied. Using OSL, the data were down-sampled to 250 Hz, and a 0.1 Hz high-pass filter was applied to remove low-frequency drift. Artefacts associated with eye blinks, eye movements, and heartbeat were rejected using independent-component analysis. Between 0 and 3 components were removed for each participant; on average 2.2 components were removed. All artefactual components were inspected visually before removing them from the data. OSL’s variance-based automated artefact detection was applied after epoching of the data to exclude trials with excessive noise. This method uses robust regression (Holland and Welsch, 1977) to fit the average variance across epochs whilst down-weighting trials which are particular influential to the results. Trials whose final weighting was below 0.2 were removed from subsequent analyses. Trials excluded during the behavioural analysis stage were excluded from the MEG data as well. On average 5.2 ± 0.6 % of trials per participant were excluded from the analysis (5.5 ± 0.9 % from control participants; 5.0 ± 0.8 % from PD participants).

#### 2.5.2 Analysis of event-related fields for region-of-interest selection

Analysis of event-related fields (ERFs) was performed using MATLAB and Fieldtrip (Oostenveld et al., 2011). Two motor regions-of-interest (ROIs) were selected based on ERFs locked to all left- and right-hand button-presses. To avoid potential circularity in the analysis, this ERF-based ROI selection was independent of the primary time window of interest (the pre-target, anticipatory, period), as well as of the signal feature of interest (beta-band activity), and the condition-comparison of interest (anticipatory dynamics following early vs. late cues).

**Fig. 3:**
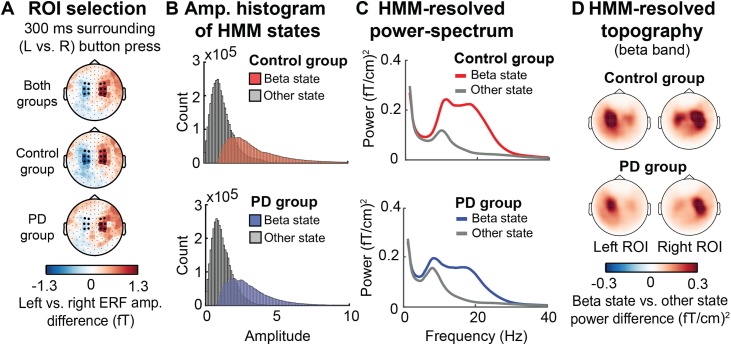
**Basic HMM characteristics.** (A) Topographies for the left-minus-right response amplitude difference in the ERF in a ± 150-ms window relative to the button press (averaged for early and late targets), using a standard evoked-response analysis. The six left and six right central-motor channel pairs that were used as ROIs throughout the subsequent MEG analyses are marked in black. Channels were selected based on inspection of the ERF grand average for all participants. Separate topographies for the control (middle plot) and PD (bottom plot) groups show that the largest ERF amplitudes were found in the same channels for both groups. (B) Amplitude histograms of HMM states, separately for the control group (top) and PD group (bottom). The beta state is shown in colour, while the other state is shown in grey. (C) The HMM-resolved state-specific power spectra for contralateral motor channels for the control group (top panel) and PD group (bottom panel). (D) HMM-resolved topographies for the difference between the beta state vs. the other state, averaged across the beta band (15−28 Hz).

ERFs were calculated separately for left and right responses. Data for the planar gradiometer pairs were combined (cartesian sum), resulting in a 102-channel combined planar gradiometer map in sensor space. A left vs. right difference ERF was calculated for each participant separately and subsequently averaged across participants. Motor ROIs were selected based on the left vs. right difference ERF topography in a ± 150 ms window relative to the button press, from the grand average across all participants. Based on the topography, six symmetric channel pairs were selected. Left sensors were MEG0412 + 0413, MEG0422 + 0423, MEG0432 + 0433, MEG0442 + 0443, MEG1812 + 1813 and MEG1822 + 1823; and right sensors were MEG1112 + 1113, MEG1122 + 1123, MEG1132 + 1133, MEG1142 + 1143, MEG2212 + 2213 and MEG2222 + 2223. The distribution of response-related ERFs was similar for both groups, so that similar channels would have been selected based on the averages for each group separately (see Fig. 3).

#### 2.5.3 Time-frequency analysis

Time-frequency analysis was performed using MATLAB and Fieldtrip (Oostenveld et al., 2011). Before evaluating beta-band modulations using the Hidden Markov Model (described in more detail below) we first performed a conventional time-frequency analysis using a short-time Fourier Transform and a Hanning taper for frequencies between 4 and 45 Hz (in 0.5 Hz steps). A fixed sliding time window of 300 ms was advanced over the data in steps of 50 ms. Time-frequency power values were combined for planar gradiometer pairs to get a 102-channel combined planar gradiometer map. For each individual we then averaged power values for the subject-specific beta-band (see Supplementary Fig. 1), separately for contralateral left and right motor ROIs and for short and long cues. The resulting data were baselined with the 200-ms period before cue appearance (calculated as a relative change: ((data - baseline)/baseline × 100). For our main analysis on the early window, all trials were included, independent of cue validity. For our analysis of the post-early target window (presented in the Supplementary Materials), invalidly cued targets and NoGo targets had to be excluded.

Beta-power time courses for our different experimental conditions were evaluated statistically by means of temporal-cluster-based non-parametric permutation testing in Fieldtrip (accounting for multiple-comparisons along the time axis; Maris and Oostenveld, 2007). We used 1000 permutations with a (two-sided) cluster alpha of 0.05. Reported cluster p-values reflect one sided cluster p-values unless stated otherwise.

In both groups, the relationship between the behavioural results and beta power (in the last 200 ms of the early window) was evaluated in a correlational analysis. In addition, in the PD group the relationship between UPDRS-III scores and beta power was examined.

#### 2.5.4 Hidden Markov Model (HMM) analysis

The HMM can represent oscillatory dynamics as a time course of discrete state visits (see Quinn et al., 2018 for an introduction to the HMM and its use in MEG; see also Baker et al., 2014; Vidaurre et al., 2016). We used the HMM to describe amplitude dynamics in the beta band, and, more specifically, how visits to a high beta-amplitude state changed with our task conditions and differed between our PD and control groups. This provides an elegant approach for event-based characterisation of neuronal dynamics (to help distinguish among the various scenarios that may account for condition differences in trial-average beta power; Fig. 1). The HMM approach bypasses the need to set an arbitrary (user-defined) threshold for classifying “states” of high beta amplitude, and encompasses temporal regularisation to avoid that periods of high amplitude are split into many small events due to small noisy changes in amplitude close to a given threshold.

Prior to HMM inference, individual beta-band amplitude envelopes were calculated separately for left and right motor ROIs by bandpass-filtering sensor space data around the individual beta peak ±6 Hz (see Frequency analysis and individual beta peak detection). These averages were downsampled to 100 Hz, and the Hilbert transform was used to compute the amplitude envelope.

In previous work, the HMM has typically been used to identify multiple states, where each state corresponds to a different large-scale brain network (Baker et al., 2014; Quinn et al., 2018; Vidaurre et al., 2018a,b). Here, we instead intend to use the HMM to identify beta events at a single spatial location at a time. We therefore inferred a two-state Amplitude Envelope HMM (AE-HMM; see Baker et al., 2014; Hunyadi et al., 2019; Quinn et al., 2018) to describe dynamics in the beta-band amplitude envelopes, with a separate HMM being inferred for the left and right motor channels and for each individual. The HMM analysis was performed using the HMM-MAR toolbox (https://github.com/OHBA-analysis/HMM-MAR). Within the HMM, each state can be described by a binary state time-course (Viterbi path), describing when the state switches “on” or “off”, and a Gaussian distribution describing the amplitude values observed whilst the state is on. This observation model describes the amplitude values across all time-points from all visits to the state. Note that the HMM is inferred using stochastic inference (Vidaurre et al., 2018a,b) on the continuous data, and with no knowledge of the task structure. To ensure that the HMM results were stable across multiple runs of the inference, we repeated the HMM ten times for each participant for both left and right ROIs, after which we selected the run with the lowest value of free-energy (as described in Quinn et al., 2018).

Building on previous work using fixed amplitude thresholds, we inferred the HMM by deliberately specifying two states for each dataset, so the HMM would label each time point within each participant’s time course as either high or low amplitude beta. We refer to the state with the higher mean amplitude value in its observation model as the as the “beta state”. The state with the lower mean amplitude is referred to as the “other state”. As the two states are mutually exclusive, their dynamics are the inverse of one another. When state one is “on”, state two must be “off”. In addition, the duration of a visit to one state is also the interval between visits to the other. As such, we can focus only on the dynamics of the beta state in which “on” periods are high-amplitude events. As well as the Viterbi path, the HMM can provide the posterior probability of being in a given state for each point in time, known as the Gamma time-course. In contrast to the Viterbi path, the Gamma variables vary parametrically between zero and one. Several metrics are computed from these time-courses to explore whether task-related changes in beta power arise from the duration, frequency or amplitude of these discrete events.

Firstly, the beta-state fractional occupancy time course represents the probability of being in the beta state at each moment in time during a trial. This was calculated for each time point in each experimental condition by epoching and averaging the Gamma variable separately for trials following cues predicting short vs. long motor-preparation intervals.

In addition to the time course of fractional occupancy, we calculated time courses for a) beta-state amplitude b) beta-state lifetime and c) beta-state interval time. A state-specific amplitude time-course was computed by taking the state time-course (containing zeros when the state is “off” and ones when the state is “on”) and replacing the values during each visit with the amplitude of that visit. The resulting time source contains zeros when the state is “off” and the amplitude of each visit when the state is on. Amplitude values were taken from the individual beta-band amplitude envelopes (the HMM input). Similarly, to calculate beta-state lifetime time courses, each state assignment was replaced with the particular duration of that beta-state visit (while visits to the other state were ignored). For the interval time we replaced each Viterbi assignment to the other state (i.e. when the beta state was considered more likely to be “off” than “on”) with the duration of that particular interval (and ignored the visits to the beta state). Finally, time courses were epoched and averaged for each experimental condition as described above.

Cluster-based non-parametric permutation testing was used to evaluate differences between the two movement preparation conditions (expect late vs. expect early) and between the two groups for the beta-state fractional occupancy, beta-state amplitude, beta-state lifetime and beta-state interval time. In addition, the relationship between the behavioural results and HMM measures and between clinical symptoms in the PD group and HMM measures were evaluated in a correlational analysis (see Supplementary Analysis 5).

### 2.6 Data availability

The authors will make the data available upon reasonable request.

## 3 Results

### 3.1 Effect of temporal cues on motor performance is diminished in Parkinson’s disease

We first assessed both groups in terms of their overall RTs. RT distributions for all participants (collapsed across conditions) are plotted in Fig. 2B, with control participants in red and PD participants in blue. The bottom panel shows group averages and reveals that RT distributions were highly comparable between groups. We compared average RTs and variance in RT between both groups with an independent-samples t-test. RTs were not significantly different between both groups (control group: M ± SD = 427 ± 67 ms; PD group: M ± SD = 424 ± 41 ms; t(34) = 0.174, p = .863) and the same was true for the variance (t(34) = 0.447, p = .658).

We next analysed RT as a function of our temporal cues, as shown in Fig. 2C. To assess the influence of the temporal motor-preparation cues, we statistically compared effects on early-target trials, given that a large body of research has shown that temporal cueing effects are largely restricted to the early target (see e.g. Coull and Nobre, 1998; Miniussi et al., 1999; Nobre, 2010; Rohenkohl et al., 2014; Heideman et al., 2018; also reviewed in Nobre and van Ede, 2018) – though we present the mean ± SE of all experimental conditions in Supplementary Table 2. A repeated-measures ANOVA with the factors Cue Validity and Group showed a main effect of Cue Validity (F(1,34) = 29.86, p < .0001, partial η2 = 0.47) and a significant interaction between Group and Cue Validity (F(1,34) = 5.46, p = .026, partial η2 = 0.14). There was no main effect of Group (F(1,34) = 0.08, p = .78, partial η2 = 0.002). Post-hoc pairwise t-tests revealed that for both groups a validity effect was present (control participants: t(17) = 5.35, p < .0001, Cohen’s d = 1.78; PD participants: t(17) = 2.29, p = .035, Cohen’s d = 0.76), and that this effect was larger in control participants than in PD participants: t(34) = 2.42, p = .021, Cohen’s d = 0.81 (see also Fig. 2D). Thus, while both groups were faster to respond following valid vs invalid temporal cues, this temporal validity effect was diminished in PD participants.

Accuracy in this simple task was uniformly high across temporal motor-preparation condition and group. A statistical analysis was performed for the percentage correct in Go trials and NoGo trials. These data are shown in Supplementary Table 2. For Go trials, this analysis showed no main effect of Cue Validity (F(1,34) = 0.01, p = .93, partial η2 < 0.001), no main effect of Group (F(1,34) = 0.13, p = .73, partial η2 = 0.004), and no interaction between Cue Validity and Group (F(1,34) = 0.01, p = .93, partial η2 < 0.001). Similarly, for NoGo trials, this analysis also showed no main effect of Cue Validity: F(1,34) = 2.56, p = .12, partial η2 = 0.07), no main effect of Group (F(1,34) = 0.87, p = .36, partial η2 = 0.03), and no interaction between Group and Cue Validity (F(1,34) = 0.01, p = .91, partial η2 < 0.001).

### 3.2 Region-of interest selection

Motor ROIs (Fig. 3A ) were selected after inspection of ERF topographies for the difference between left vs. right responses in a ± 150 ms window centred at the button press (collapsed over responses following early and late targets). ROI selection was performed on the grand average across all participants, selecting the six (symmetrical) left and right channel pairs with the largest left vs. right ERF amplitude difference. Importantly, the same sensors would have been selected based on either the control or PD group in isolation (see Fig. 3A).

### 3.3 The Hidden Markov Model enables blind extraction of beta state with meaningful profiles

Before turning to our primary research question, we first evaluated the validity of the beta-state allocation by the HMM pipeline. To this end, we investigated amplitude histograms associated with both states, and also revisited the raw data during each state to map the spectral profiles and topographies that correspond to the HMM-allocated beta state.

Amplitude histograms associated with the HMM-derived beta state and the other state are shown in Fig. 3B, separately for participants in the control group (top) and the PD group (bottom). Separate histograms of beta amplitude were computed for the beta-state and other-state portions of the data based on the Viterbi path, which contains an exclusive state assignment to either the beta state or the other state for each point in time. This figure shows that there is a clear differentiation between both states, with most higher amplitudes assigned to the beta state and most lower amplitudes assigned to the other state (note that this state assignment was purely data driven, without a fixed amplitude threshold as in previous approaches to isolate beta “events”; e.g. Feingold et al., 2015; Lundqvist et al., 2016; Shin et al., 2017; Tinkhauser et al., 2017a, 2017b; 2018). This figure shows that the HMM was able to separate both states as expected. Note that there is some overlap between both states because the HMM assigns states in a temporally regularised manner, meaning that surrounding time points play a role in determining the state assignment for each point in time.

The HMM-resolved power spectrum for the contralateral ROI is shown in Fig. 3C, separately for the control group (top panel) and the PD group (bottom panel). This full frequency spectrum for each state was computed by weighting the raw data at each time point by the posterior state probabilities (the Gamma HMM output variable) and subsequently calculating the frequency spectrum using a multitaper approach (Vidaurre et al., 2016; Quinn et al., 2018). This figure shows that, as expected, beta-band activity is mostly captured by the beta state in both groups.

Finally, HMM-state dependent topographies are shown in Fig. 3D. This figure depicts the difference between the beta state and the other state in the beta band when the HMM results are projected back to all channels for the full frequency spectrum from 0−40 Hz (as described above - but now for all channels) and subsequently averaged for the beta band (15−28 Hz). Results are shown separately for the left and right ROI (for which separate HMMs were performed) for both groups, with the control group shown at the top and the PD group shown at the bottom. These results show that beta-state visits are to a large extent unilateral and specific to the central (putative motor) channels of interest.

In addition to these checks, we also compared average beta-state durations (“lifetimes”) and the number of beta-state visits across the whole continuous dataset between groups. Average lifetimes were 159 ± 14 (SD) ms in the control group and 150 ± 10 ms in the PD group, which is similar to lifetimes reported previously in e.g. Baker et al. (2014) and Quinn et al. (2018). The group difference was significant (t(34) = 2.7, p = .01). However, the total number of beta-state visits (i.e. the rate) was not different between groups (t(34) = -1.41, p = .168).

Together, these “checks” confirm that the HMM yielded a sensible allocation of beta states, that are characterised by high amplitude, by spectra that are dominated by beta frequencies, and by a motor-channel-centred topography.

### 3.4 HMM fractional occupancy recovers beta-power decreases during timed movement preparation

**Fig. 4:**
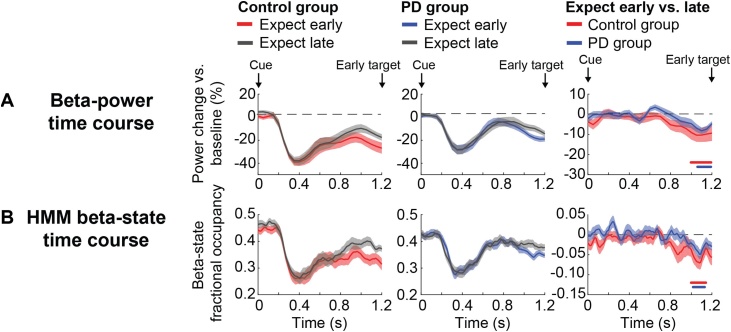
**Beta power and HMM fractional occupancy during movement preparation in the anticipatory window.** Results following a (A) conventional time-frequency analysis and (B) HMM analysis on beta-band changes over time. (A) Beta-power changes over time (vs. pre-cue baseline) shown from cue onset (time 0) until early target presentation (1.2 s). The left panel shows results for the control group, with the expect-early (prepare for a response at 1.2 s) condition plotted in red and the expect-late (prepare for a response at 2.2 s) condition plotted in grey. The mid panel shows results for the PD group, with expect early in blue and expect late in grey. The right panel shows the expect early vs. late contrast for the control group (red) and PD group (blue). Horizontal lines indicate significant clusters after non-parametric cluster-based permutation testing (control group: p = .011; PD group: p = .005). There was no significant difference in the expect early vs. late difference between both groups. (B) Similar results following HMM analysis for beta-state fractional occupancy, which represents the likelihood of being in the beta state at a given point in time. Significant clusters are again indicated by horizontal lines (control group: p = .005; PD group: p = .02).

In our MEG data analysis, we focussed on the interval between the onsets of the cue and the early target (0–1.2 s from cue onset), because this is the window where expectations about the time of target occurrence differ, following early vs. late cues (see Coull and Nobre, 1998; Miniussi et al., 1999; Nobre, 2010; Rohenkohl et al., 2014; Heideman et al., 2018; Nobre and van Ede, 2018). Before using the HMM to quantify changes in the characteristics of beta-state visits, we first wanted to establish the general pattern of beta-power modulation over time, by performing a conventional time-frequency analysis. Fig. 4A shows beta-power time courses for the expect-early and expect-late conditions for the control group (left panel) and PD group (middle panel), as well as for the difference between expect early and expect late (right panel) for both groups overlaid. In both groups, beta power is lower in the expect-early compared to the expect-late condition, reaching significance in the time period starting approximately 200 ms before the end of the anticipatory window (control group: cluster p = .011; PD group: cluster p = .005). However, the expect early vs. late difference did not differ significantly between both groups.

As explained before, the primary reason for using the chosen HMM approach was to interrogate the single-trial dynamics (“state parameters”) that resulted in the trial-average beta-power decrease as observed in the conventional analysis of power. Before turning to this, however, we first wanted to ascertain that the time course of our HMM beta state was indeed also sensitive to picking up this trial-averaged power decrease.

To this end, we calculated the time course of the HMM fractional occupancy (see Fig. 4B) which represents the probability of being in the beta state over time. As expected (because we performed a two-state HMM based on amplitude), this variable followed a similar pattern to what we saw in Fig. 4A for power, with a significant difference in the expect early vs. expect late condition starting ∼200 ms before potential early target appearance (control group: cluster p = .005; PD group: cluster p = .02). The group difference was again not significant. Thus, both beta-state fractional occupancy and beta power decreased during movement preparation for upcoming targets.

### 3.5 Increased interval time between beta-state visits accounts for overall power decrease and distinguishes PD group from control group

**Fig. 5:**
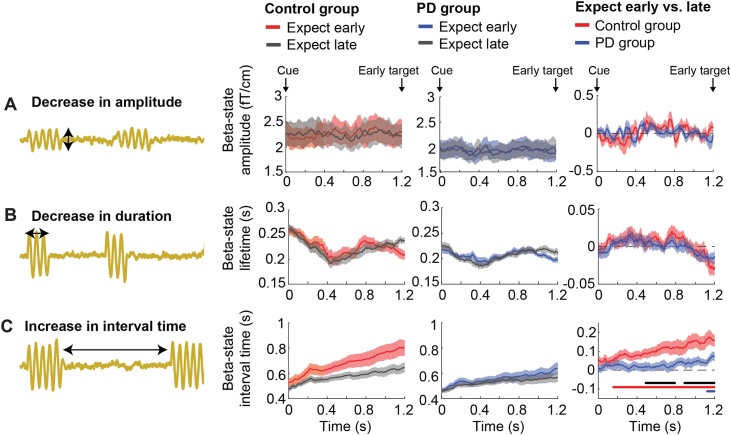
**Beta-state amplitude, lifetime and interval time in the anticipatory window**. Schematic in yellow shows possible mechanisms of (beta) power decrease. The right plots show results for (A) beta-state amplitude; (B) beta-state lifetime; (C) beta-state interval time. Time courses are presented for early and late target expectations following temporal cues. Left panels show data for the control group for early (red) and late (grey) expectations. Middle panels show data for the PD group for early (blue) late (grey) expectations. Right panels show the expect early vs. late difference for the control group (red) and the PD group (blue). Shaded areas reflect standard error of measurement. Horizontal bars show significant clusters after temporal-cluster-based non-parametric permutation testing for control participants (red; cluster p = .002) PD participants (blue; cluster p = .049) and the group difference (black; early cluster p = .024; late cluster p = .023).

As highlighted in Fig. 1, the HMM allows us to investigate what aspects of the beta-state occurrence change during movement preparation, by interrogating changes in beta-state characteristics that can be computed from the HMM outcomes. As Fig. 1 showed, there are at least three possible causes for the decreases in beta power that we see in Fig. 4A, and which can be distinguished from the HMM results: a decrease in beta-state amplitude, a decrease in beta-state lifetime, and an increase in the beta-state interval time (corresponding to a decrease in the rate of occurrence of beta-state visits). To help guide interpretation of our key findings, we depicted these three possibilities again on the left of all relevant panels in Fig. 5. For each variable, the HMM results are shown for the expect early and expect late conditions for the control group (left panel) and the PD group (middle panel) and for the early vs. late difference (right panel).

Fig. 5A shows that beta-state amplitude is relatively constant and is not significantly or detectably modulated by our experimental conditions. Beta-state lifetime/duration (Fig. 5B) shows an initial decrease (0-0.4 s post cue-onset) followed by an increase (>0.4 s). However, this was not significantly or detectably modulated by our experimental conditions. In contrast, the beta-state interval time (Fig. 5C) shows a gradual increase from the ∼0.2 s post cue-onset, corresponding to a gradual decrease in the rate of occurrence of beta-state visits (i.e. beta events; see Supplementary Fig. 2 for the average number of beta events in the 0.4–1.2 s window for each condition for each group). This was significantly modulated by experimental condition both in the control group and the PD group. In the control group this difference between experimental conditions already started around 0.2 s after cue presentation and kept increasing all the way to the end of the anticipatory interval (cluster p = .002). In the PD group this difference was significant for the last ∼0.1 s only (cluster p = .049).

The parameter “beta-state interval time” was not only the primary variable that showed a difference between early vs. late target expectations, but also was the only variable that showed a significant difference between both groups (early cluster: cluster p = .024; late cluster: cluster p = .023), with a larger difference in the control group, compared to the PD group (in direct correspondence with the diminished behavioural validity effect of the temporal cues on RT).

These results suggest that the general decrease in beta power during the anticipatory period does not arise from beta events having a lower amplitude, but instead arises from the increased interval between beta events. This combines with an initial decrease in beta event duration (0-0.4 s post cue-onset) followed by an increase (>0.4 s), to produce the overall beta-state fractional occupancy and beta-power time courses in Fig. 4. In addition, these results reveal that the diminished behavioural validity effect of the cue is paired with a diminished anticipatory neural modulation in PD participants – a group difference that only became apparent in our analysis when specifically considering the beta-state-parameter that corresponds to the interval between high-amplitude beta events.

In a post-hoc analysis, we used a simpler and more conventional way of detecting beta events and recalculated our event-parameters using a median-amplitude threshold. As shown in Supplementary Fig. 3, this complementary analysis revealed an overall similar pattern of results, and again revealed a clear group difference for the modulation of inter-event interval times. However, using this simpler thresholding approach, we no longer observed significant differences between the expect early vs. late conditions in the PD group for any of the analysed parameters. Thus, while this analysis corroborated our most important observations, it appears less sensitive than our HMM-based analyses.

### 3.6 Correlations with behaviour

**Fig. 6:**
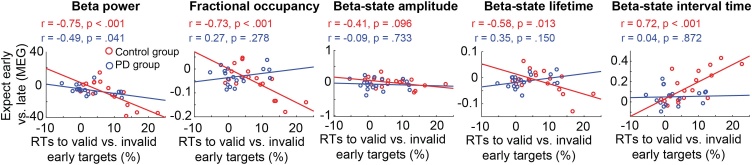
**Correlation between behavioural performance and MEG outcomes**. Correlations between behavioural performance (relative RT difference between validly cued vs. invalidly cued early targets) and the expect early vs. expect late difference in the 200 ms preceding early target presentation for beta power, HMM fractional occupancy, beta-state amplitude, beta-state lifetime, and beta-state interval time for control participants (red) and PD participants (blue). r values reflect the Pearson correlation coefficient.

To investigate the behavioural relevance of the expect early vs. late differences, we performed a correlational analysis between the temporal cueing effects on behaviour and the expect early vs. late neural differences in the last 200 ms of the anticipatory window (for each of the considered parameters). This analysis is presented in Fig. 6: the x-axis shows the relative RT difference between validly vs. invalidly cued targets presented after the early interval, and the y-axis shows the neural difference between expect early and expect late during the last 200 ms preceding the early target.

For beta power, the Pearson correlation with the behavioural performance effect was significant for both groups (Control group: 2 = −0.75, p < .001; PD group: r = −0.49, p = .041) and not significantly different between both groups (tested using Fisher’s r-to-z transformation; z = -1.2 p = .115). For fractional occupancy, the correlation with behaviour was significant in the control group only (control group: r = -0.73, p < .001; PD group: r = 0.27, p = .278). Moreover, correlations were significantly different between both groups (z = -3.3, p = .0005). For the three HMM state characteristics, correlations with behavioural performance were not significant for beta-state amplitude (control group: r = -0.41, p = .096; PD group: r = -0.09, p = .733), but were significant in the control group only for beta-state lifetime (control group: r = -0.58, p = .013; PD group: r = 0.35, p = .150) and beta-state interval time (control group: r = 0.72, p < .001; PD group: r = 0.04, p = .872). As for fractional occupancy, these correlations were also significantly different between both groups (lifetime: z = -2.82, p = .002; interval time: z = 2.38, p = .009) – possibly owing to the reduced magnitude of the cueing effects on behaviour and brain dynamics in the PD group.

In addition, to investigate the influence of PD symptoms on each of our measures, we calculated correlations between UPDRS-III scores and behavioural- and MEG-derived variables. This showed no significant effects and is described in Supplementary Analysis 5.

### 3.7 Post-hoc analysis in the post-target window

Finally, to investigate if our group difference in the interval time of the beta state was specific to movement preparation, we evaluated the early vs. late difference during response execution in the post-early target window. This analysis is presented in Supplementary Fig. 4. In this window, not only interval times, but now also lifetimes tracked the time course of motor execution in the hypothesized direction (see Fig. 1) and showed a significant early vs. late difference. Lower beta power went together with both decreased lifetimes and increased interval times. In contrast, the period of higher power (beta rebound) after the response was made was accompanied by the opposite pattern (increased lifetimes and decreased interval times). In contrast to the anticipatory window, there was no group difference for the early vs. late response contrast. Beta-state amplitude was again consistent over time and did not show a difference between early and late responses.

## 4 Discussion

This study investigated changes in high-amplitude beta events during temporally-cued movement preparation in Parkinson’s disease. We show that beta-state interval time is the main variable that increases between stronger vs. weaker movement preparation, providing a more mechanistic insight into beta activity during task performance than accounts that focus on beta power alone. Furthermore, we show that this variable is a sensitive marker, because changes in the interval time between successive beta events were reduced in PD participants compared to healthy control participants. In both groups, high-beta events became rarer with increasing movement preparation, but this effect happened less strongly and started later in the PD group, congruent with the smaller behavioural effect in this group.

Beta-band activity is generally found to decrease prior to and during movement, which is followed by a sharp increase after movement offset (Pfurtscheller and Lopes da Silva, 1999; Kilavik et al., 2013). Although the precise functional significance of beta-band activity in the motor system is still unclear, it is generally agreed that cortical beta suppression is an indicator of motor readiness (Jenkinson and Brown, 2011) and that also in other contexts beta may reflect a passive or more active inhibitory process (Hari and Salmelin, 1997; Engel and Fries, 2010). However, the emerging perspective proposing the existence of beta events (Feingold et al., 2015; Jones, 2016; Lundqvist et al., 2016; Sherman et al., 2016; Shin et al., 2017; Tinkhauser et al., 2017a, 2017b; 2018; van Ede et al., 2018; Little et al., 2019) enables finer grained descriptions of modulation of frequency-specific patterns of brain activity, with implications for our understanding of their origin, and functional relevance.

Increases in the beta-state interval time correspond to decreases in the rate of occurrence of beta events. Similar to the current findings, Shin et al. (2017) recently found that beta event-rate in somatosensory cortex was the best predictor of both tactile stimulus detection and shifts in attention, and that this variable correlated more strongly with pre-stimulus beta power than event power, event duration or event frequency span. Likewise, Little et al. (2019) found a similar inhibitory relationship between beta-event occurrence and behaviour in the context of a motor task, with faster responses being associated with a lower pre-movement event rate. In line with this inhibitory relationship between the occurrence of beta events and task behaviour, we found that the interval between subsequent beta visits was larger when participants anticipated having to respond early, compared to when they anticipated having to respond late. Furthermore, we found that this neural effect strongly correlated with behavioural performance effect, at least in the control group (for which both effects were more pronounced). In other words, control participants with a strong anticipation effect in interval times (i.e. a strong decrease in beta-state occurrence) also showed strong effect of temporal anticipation in behaviour.

In the PD group the effects of temporal anticipation were smaller, both in the HMM results and in behaviour. An important open question remains as to whether this reflects a reduced ‘ability’ or a reduced ‘motivation’ to use the temporal cues to prepare movements in advance. Still, these effects are unlikely to be due to differences in overall task-engagement between both groups, as we confirmed that overall RT, RT variability, and accuracy were highly similar between both groups.

In addition to giving a more detailed, mechanistic description of the single-trial constituents of beta-activity suppression during timed movement preparation, our approach also revealed that such single-trial characterisation of anticipatory brain dynamics enabled more sensitive group comparisons, in this case between PD patients and control participants. Significant group differences could not be established from looking at beta-power changes alone, but were evident when focusing on the identified relevant event parameter.

Our results primarily reveal the practical advantages of disentangling the various single-trial parameters that can contribute to changes in trial-averaged power. Using the HMM approach, it was possible to uncover which single-trial parameters were modulated during timed movement preparation and to charter group differences in a more fine-grained and sensitive manner. However, our data do not directly speak to the physiological mechanisms underlying the putative beta events, nor do they reveal whether and how such events may be used in neural computations (for example, whether events are read out in downstream populations as a ‘rate code’). Indeed, as we have previously argued (van Ede et al., 2018), it is notoriously difficult to discern the appropriate physiological interpretation of frequency-specific patterns of MEG activity. Nevertheless, by using the HMM to quantify single-trial event parameters that can lead to changes in trial-averaged power, we have shown how it is possible to characterise the nature of these changes with enhanced granularity as well as to capture group differences in these changes with increased sensitivity. These are the primary contributions of the present work.

An obvious question is why do the differences that appear in the beta-state interval times (Fig. 5C) do not produce corresponding differences in the beta-state fractional occupancy time courses (and beta-power time courses) in Fig. 4B? This is likely to be because the HMM fractional occupancy time courses are produced from a combination of not only the beta-state interval time, but also the beta-state lifetime (duration). In this case, the beta-state lifetime starts to increase > 0.4 s post cue-onset, thereby counteracting the gradual increase in interval time, masking its effect on the beta-state fractional occupancy. This highlights the utility of the more detailed measures of the neuronal dynamics provided by the HMM analysis.

Several other recent studies have already investigated beta-event dynamics in Parkinson’s disease, but these mainly relied on invasive recordings during rest. For example, local field potentials from the basal ganglia in PD patients showed pathological prolongation of high-amplitude beta events, the extent of which correlated with motor impairment (Tinkhauser et al., 2017a, 2017b; see also Feingold et al., 2015). It was further shown that event durations decreased with levodopa treatment and adaptive deep-brain stimulation (Tinkhauser et al., 2017a, 2017b). Instead, in our study, we focused on anticipatory beta dynamics during timed movement preparation in extracranial MEG measurements. In contrast to the subcortical findings by Tinkhauser et al., in our study, events (irrespective of task condition) were, on average, shorter in PD participants than in control participants. However, studies looking at cortical motor beta power measured using M/EEG, often report similar (de Hemptinne et al., 2013) or reduced beta power in PD compared to control participants (Bosboom et al., 2006; Stoffers et al., 2007; Vardy et al., 2011). Thus, changes in cortical beta in PD may stand in contrast to the excessive beta-oscillatory synchrony found in basal-ganglia (see e.g. Hammond et al., 2007; Little and Brown, 2014; Oswal et al., 2013, but see also Pollok et al., 2012; Crowell et al., 2012).

Overall, we show that the HMM is a powerful method for characterising changes in beta events that lead to changes in trial-averaged beta power and that are related to behaviour. Our data also make clear that this method is promising for studying changes in neurodegenerative disorders affecting beta. This method could potentially have powerful implications for understanding the nature of beta-power changes, and their cognitive modulations in a variety of clinical conditions. Applying the approach as we have taken here to other clinical populations will be an important target for future inquiry.

## Declaration of Competing Interest

The authors declare no competing financial interests.
