# Supplementary material for: Dissecting beta-state changes during timed movement preparation in Parkinson’s disease
Source: Prog Neurobiol. 2020 Jan;184:101731. doi: 10.1016/j.pneurobio.2019.101731 (PMC6977086; doi:10.1016/j.pneurobio.2019.101731)
Supplement: Supplementary file 1 [file mmc1.docx]

**Supplementary Material**

**Dissecting beta-state changes during timed movement preparation in Parkinson’s disease**

Simone G. Heideman^1^, Andrew J. Quinn^1^, Mark W. Woolrich^1^, Freek van Ede^1^* & Anna C. Nobre^1,2^*

* These authors contributed equally

*^1^Oxford Centre for Human Brain Activity, Wellcome Centre for Integrative Neuroimaging, Department of Psychiatry, University of Oxford, Oxford, United Kingdom*

*^2^Department of Experimental Psychology, University of Oxford, Oxford, United Kingdom*

**Corresponding author**

Anna Christina Nobre – kia.nobre@ohba.ox.ac.uk

Oxford Centre for Human Brain Activity

Wellcome Centre for Integrative Neuroimaging

Department of Psychiatry

Warneford Hospital

OX37JX, Oxford

United Kingdom

**Supplementary Table 1**

*Supplementary Table 1.* Clinical details of PD participants

| Nr. | Gender | Age | Handedness | LS | HY | UPDRS-III | YSD | LEDD |
| --- | --- | --- | --- | --- | --- | --- | --- | --- |
| 1 | F | 68 | R | L | 3 | 49 | 2 | 400 |
| 2 | M | 67 | R | R | 2 | 51 | 2 | 400 |
| 3 | F | 63 | R | L | 2 | 23 | 1 | 300 |
| 4 | M | 68 | R | R | 1 | 43 | 2 | 400 |
| 5 | M | 73 | R | L | 2 | 48 | 2 | 300 |
| 6 | M | 54 | R | L | 1 | 15 | 2 | n.a. |
| 7 | M | 59 | L | R | 1 | 17 | 2 | 400 |
| 8 | M | 67 | R | L | 2 | 48 | 4 | 900 |
| 9 | M | 72 | R | R | 2 | 30 | 1 | 300 |
| 10 | F | 62 | R | R | 3 | 37 | 4 | 380 |
| 11 | M | 74 | R | L | 2 | 27 | 4 | 900 |
| 12 | F | 73 | R | L | 2 | 23 | 4 | 887.5 |
| 13 | F | 77 | R | L | 1 | 30 | 4 | 750 |
| 14 | F | 65 | R | L | 1 | 11 | 4 | 600 |
| 15 | F | 75 | L | L | 1 | 19 | 2 | n.a. |
| 16 | F | 66 | R | R | 2 | 32 | 2 | 320 |
| 17 | M | 79 | R | R | 2 | 21 | 4 | 300 |
| 18 | M | 71 | R | L | 2 | 35 | 4 | 450 |

LS: lateralisation of symptoms, most affected side; HY: Hoehn & Yahr stage; UPDRS-III: Unified Parkinson’s Disease Rating Scale subsection III score; YSD: Years Since Diagnosis; LEDD: Levadopa Equivalent Daily Dose in mg/day. n.a.: measure not applicable.

**Supplementary Table 2**

*Supplementary Table 2.* Reaction times and percentage correct for Go and NoGo trials for the control group and the PD group for all experimental conditions.

|  | Control group (Mean ± SE) | PD group (Mean ± SE) |
| --- | --- | --- |
| Reaction times |  |  |
| Early target (valid cue) | 427 ± 71 ms | 430 ± 41 ms |
| Early target (invalid cue) | 457± 64 ms | 442 ± 47 ms |
| Late target (valid cue) | 425 ± 67 ms | 418 ± 42 ms |
| Late target (invalid cue) | 431 ± 71 ms | 423 ± 45 ms |
| Percentage correct: Go |  |  |
| Early target (valid cue) | 98.6 ± 3.9 % | 98.1 ± 4.0 % |
| Early target (invalid cue) | 98.6± 4.0% | 98.1 ± 5.0 % |
| Late target (valid cue) | 98.4 ± 3.9 % | 97.4 ± 5.1 % |
| Late target (invalid cue) | 97.9 ± 4.6 % | 97.5 ± 5.4 % |
| Percentage correct: NoGo |  |  |
| Early target (valid cue) | 85.4 ± 14.5 % | 81.5 ± 16.2 % |
| Early target (invalid cue) | 90.7± 11.7 % | 86.1 ± 22.3 % |
| Late target (valid cue) | 84.0 ± 13.0 % | 82.9 ± 18.0 % |
| Late target (invalid cue) | 85.2 ± 18.9 % | 82.4 ± 29.2 % |

**Supplementary Figure and Analysis 1**


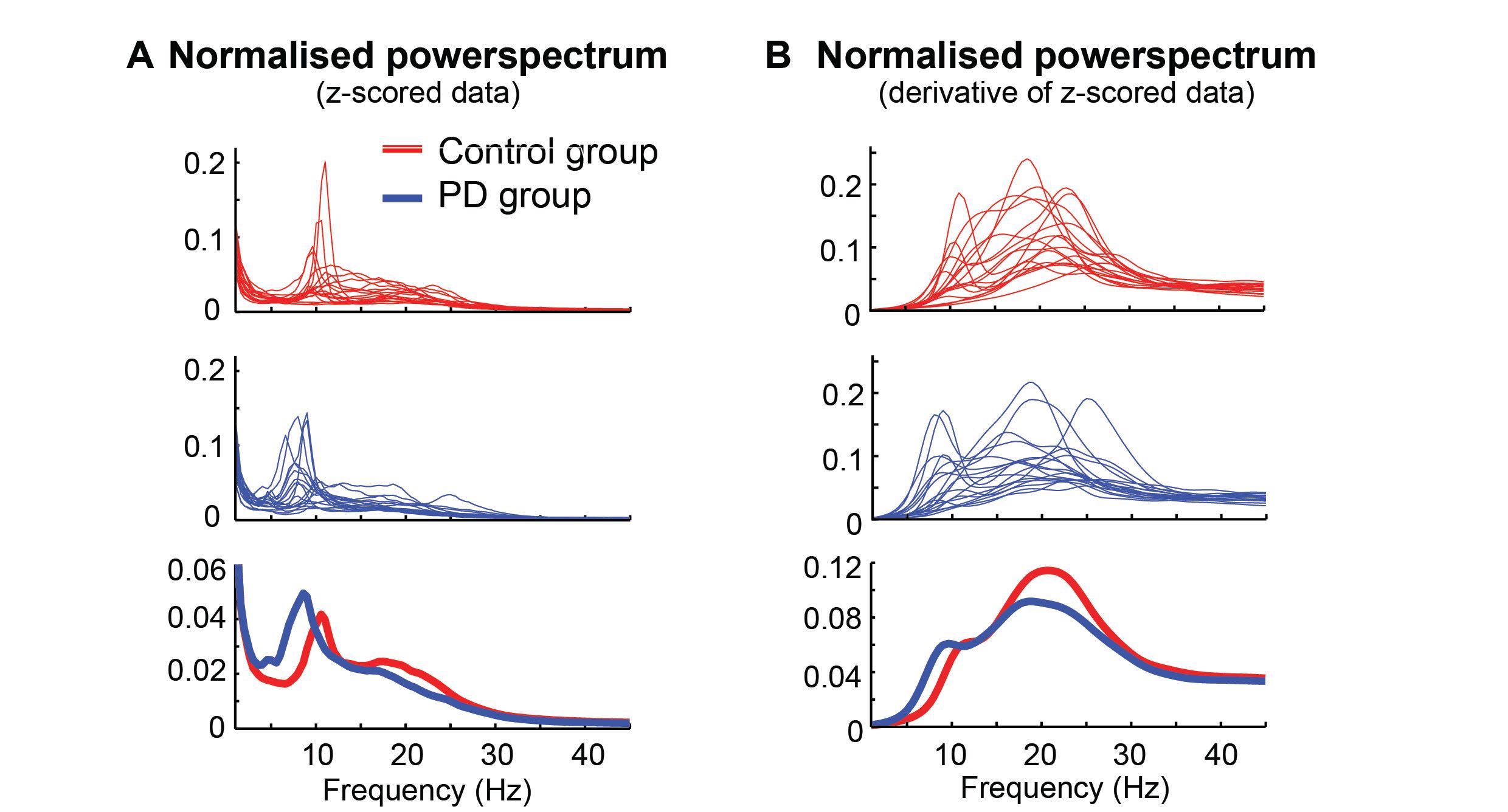


**Supplementary Fig. 1. Frequency power spectrum**. (A) The z-scored power spectrum. The top and middle figures reflect activity averaged across both motor ROIs, for a time window ranging from 0 to 2 s after cue onset, for each individual participant in the control group (top) and PD group (middle). The bottom figure shows the group average. (B) The power spectrum for data where the derivative of the signal was taken before z-scoring and performing the frequency analysis (see Time-requency analysis). The top and middle figures reflect the spectrum for each individual participant in the control group (top) and PD group (middle) and the bottom figure shows the group average. Data in (B) was used to calculate individual beta peak frequencies.

Slowing of cortical activity in Parkinson’s disease has been reported for both resting-state (e.g. Bosboom et al., 2006; Stoffers et al., 2007; Moazami-Goudarzi et al., 2008) and task data (Vardy et al., 2011). It seems to be largely unrelated to disease stage, duration, or severity; and only slightly affected by dopaminergic treatment (Stoffers et al., 2007). These potential spectral differences are important to take into account when evaluating the MEG data. Therefore, we performed our main analysis for each participant on their individual beta-band, centred on individual beta-frequency peaks.

To be able to find individual beta band peaks we performed a frequency analysis using a Fast Fourier Transform (FFT) and a Hanning taper for frequencies between 1 and 45 Hz in 0.5 Hz steps. The frequency analysis was performed on z-scored data, across all trials, for one single time window with a length of 2 s, ranging from 0 – 2 s after cue onset. Power values were combined for all planar gradiometer pairs (Cartesian sum), resulting in a 102-channel combined planar gradiometer map in sensor space. We subsequently averaged the power spectrum across all trials and across our left and right ROI channels (see Supplementary Fig. 1A).

Detection of clear beta peaks on the z-scored data was challenging for some participants and we were concerned that the detected peak in some cases may have reflected the alpha tail instead. Therefore, we repeated this analysis a second time after taking the time-domain derivative of the signal, z-scoring this data, and then performing the frequency analysis (see Supplementary Fig. 1B). A convenient feature brought about by taking the time-domain derivative of the signal is that it removes the 1/f component of the signal, while preserving the oscillatory components. This procedure allowed us to find a beta peak for every individual by taking the highest peak between 15 and 30 Hz. For the subsequent time-frequency and HMM analyses we use individual beta bands to compute participant averages, after which group averages were calculated.

**Supplementary Figure and Analysis 2**

**
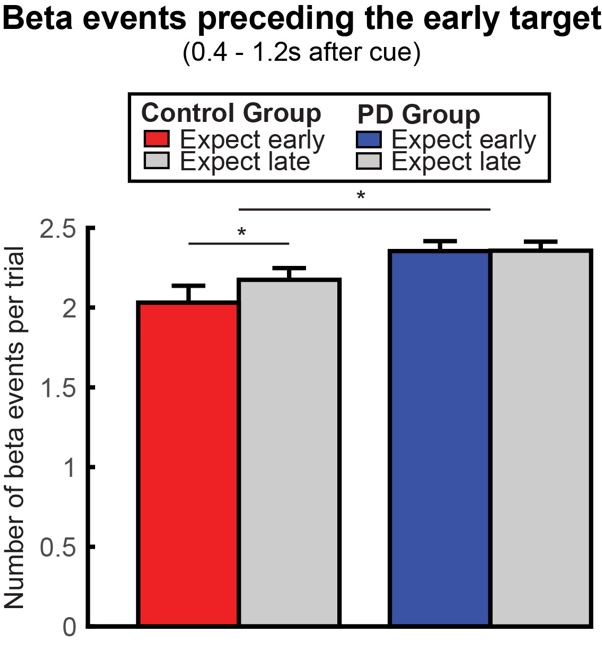
**

**Supplementary Fig. 2**. **Number of beta events preceding the early target.** The average number of beta events per trial in the window 0.4 - 1.2 s after cue onset for the control group and the PD group. Error bars show standard error of measurement (SEM). Asterisk indicate statistically significant effects.

As a complementary analysis to our analysis of beta-state lifetime, beta-state interval time and beta-state fractional occupancy, Supplementary Fig. 2 shows the average number of beta events per trial, or the beta “event rate”. For this analysis we defined beta events as each series of subsequent timepoints in the window between 0.4 - 1.2 s after cue onset where the beta state was on (and therefore the other state was off). Beta events that already started before 0.4 s or continued after target onset were included in this analysis. Note that the event rate is influenced by both the beta-state lifetime and beta-state interval time.

We performed a repeated-measures ANOVA with the within-subject factor Expectation (Early vs. Late) and the between-subject factor Group. This analysis showed a main effect of Expectation: F(1,34) = 7.098, p = .012, partial η2 = .173, a main effect of Group (F(1,34) = 5.754, p = .022, partial η2 = .145 and an interaction between Expectation and Group (F(1,34) = 6.583, p = .015, partial η2 = .162). Subsequent pairwise t-tests revealed that for the control group there was a significant difference in the number of events between expect early vs. late (control participants: t(17) = -2.890, p < .010, Cohen’s d = 0.681), while for the PD group this was not the case (t(17) = -0.116, p = .909, Cohen’s d = 0.027).

**Supplementary Figure and Analysis 3**

**
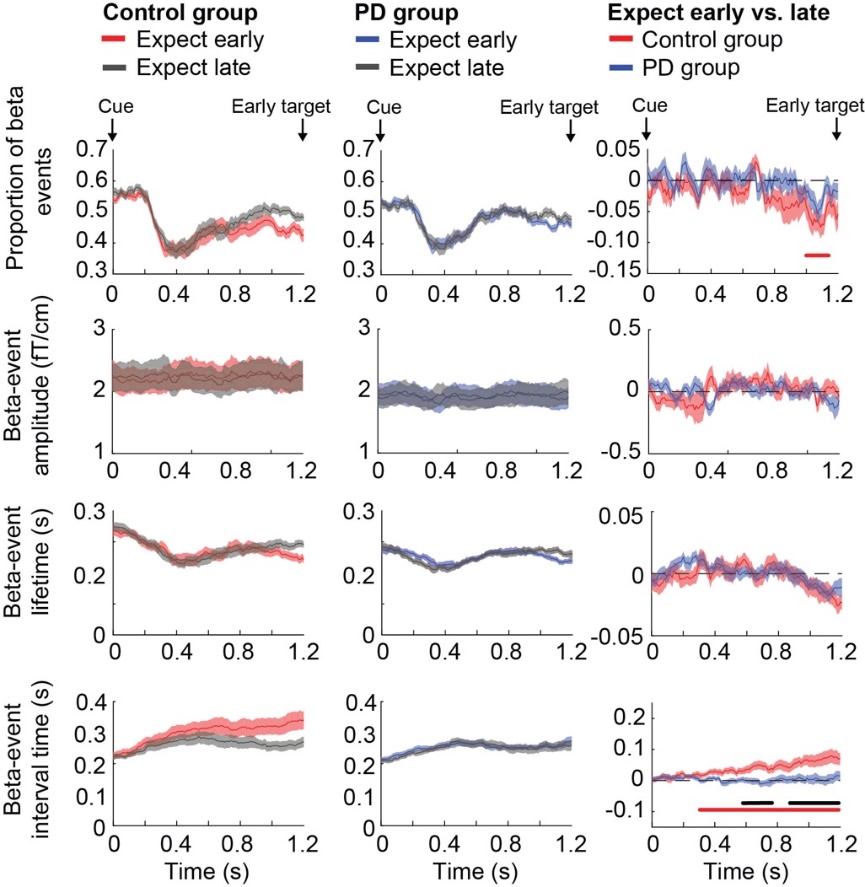
**

**Supplementary Fig. 3. Proportion of beta events, beta-event amplitude, lifetime and interval time in the anticipatory window using a median split threshold**. Results for (A) Proportion of beta events; (B) beta-event amplitude (C) beta-event lifetime; (D) beta-event interval time. Time courses are presented for early and late target expectations following temporal cues. Left panels show data for the control group for early (red) and late (grey) expectations. Middle panels show data for the PD group for early (blue) late (grey) expectations. Right panels show the expect early vs. late difference for the control group (red) and the PD group (blue). Shaded areas reflect standard error of measurement.

To see how our results compare to a simpler and more conventional approach for detecting beta events, we recalculated our main results using a median amplitude threshold, which was done for each individual on their individual beta band envelope (i.e. on the data that we used as HMM input). Data above the individual median were classified as beta events. All measures presented in Supplementary Fig. 3 are calculated the same way as their HMM counterparts, with the exception that ‘fractional occupancy’ was replaced with the ‘proportion of beta events’. While the proportion of beta events here was calculated by averaging the absolute assignment of beta events for each point in time (i.e. by averaging a series of zeros and ones), the HMM fractional occupancy is calculated from the HMM Gamma time-course (see Methods) which reflects the posterior probability of being in a given state for each point in time (i.e. by averaging a series of numbers between zero and one).

As shown in Supplementary Figure 3, though the expect early vs. late difference for the proportion of beta events (similar to fractional occupancy in the HMM analysis) is still significant in the control group (cluster p = .008), the difference no longer comes out as significant in the PD group, while in our HMM analysis this was the case. Likewise, while the interval between beta events still shows a significant expect early vs. late difference in the control group (cluster p < .001), this difference too no longer comes out as significant in the PD group. Importantly, however, just like in the HMM results, the group difference (one of our key results) is still significant (first cluster p = .042, second cluster p = .02).

The increased sensitivity of the HMM is likely to be due to the important theoretical advantage of the HMM, which is that the event dynamics are temporal regularised. In other words, a brief dip in amplitude during a period of high amplitude is not likely to lead the HMM to change to the ‘off’ state. In contrast, when using a simple threshold, small noisy changes in amplitude close to the chosen threshold can lead to a single period of high amplitude being split into many small events.

**Supplementary Figure and Analysis 4**


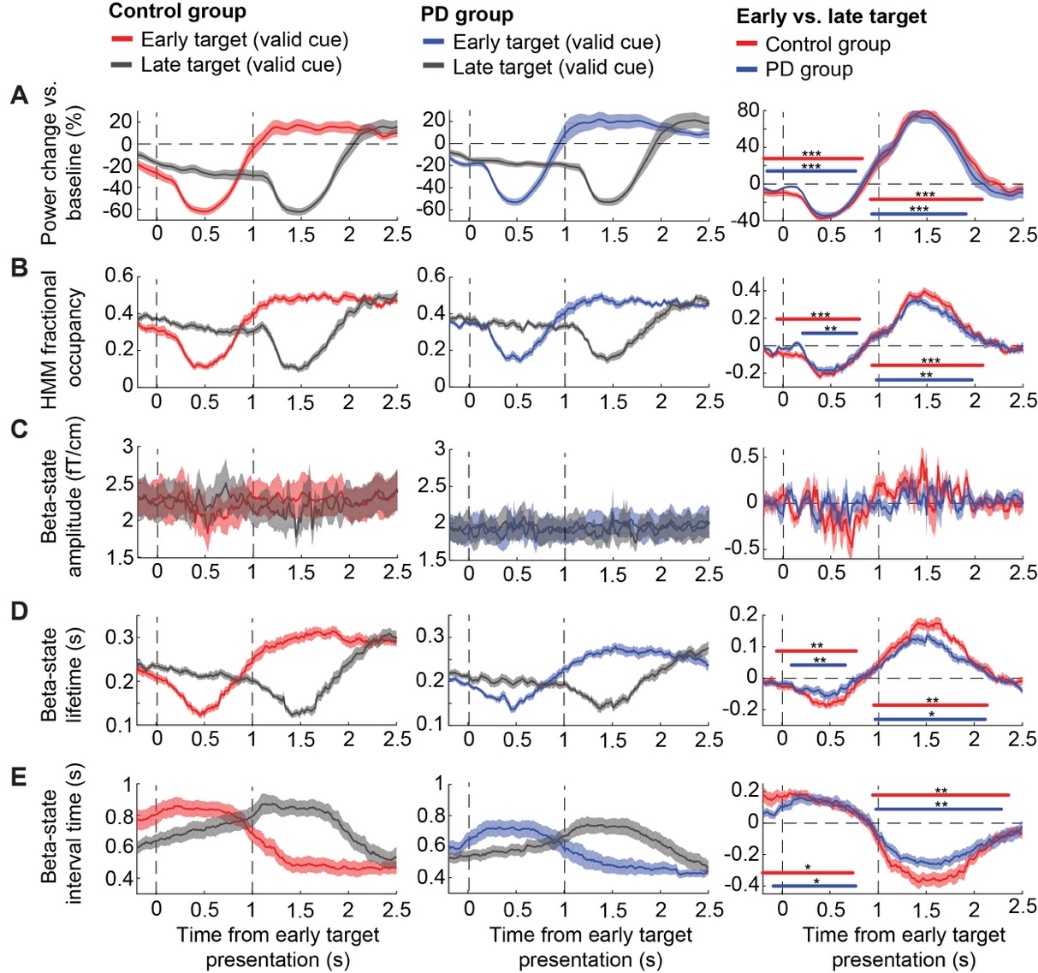


**Supplementary Fig. 4**. Changes in beta power and HMM characteristics in the post-target window. Results for (A) power change vs. pre-cue baseline; (B) HMM fractional occupancy; (C) Beta-state amplitude; (D) Beta-state lifetime; (E) Beta-state interval time. Time courses are presented for validly cued early targets (1.2 s after cue onset - here time 0) and validly cued late targets (2.2 s after cue onset - here time 1 s). Left plots show data for the control group for early (red) and late (grey) targets. Middle plots show data for the PD group for early (blue) late (grey) targets. Right plots show the early vs. late target difference for the control group (red) and the PD group (blue). Shaded areas reflect standard error of measurement. Horizontal bars show significant clusters (* p < .05; ** p< .005; *** p< .001) after cluster-based non-parametric permutation testing for control participants (red) and PD participants (blue). There were no significant differences in these effects between both groups.

The effect on beta power in the post-early target window is shown in Supplementary Fig. 5. As expected, following a target requiring a motor response beta power first drops down and then shows a strong rebound after the response was made. This effect is similar for early and late targets (but of course shifted in time based on when the target was presented). The early vs. late target difference showed two significant clusters in both groups (control group - first cluster: two-sided cluster p < .001; control group - second cluster: two-sided cluster p < .001; PD group - first cluster: two-sided cluster p < .001; PD group - second cluster: two-sided cluster p < .001). There was no significant difference between both groups.

Like in the preparatory window, beta-state fractional occupancy followed a similar pattern to beta power, reaching significance at similar times. (control group - first cluster: two-sided cluster p < .001; control group - second cluster: p < .001; PD group - first cluster: two-sided cluster p = .004, PD group - second cluster: two-sided cluster p = .002). There was again no significant group difference for this effect.

We continued by investigating the HMM effects in terms of beta-state amplitude, beta-state lifetime and beta-state interval time. As in the anticipatory interval, beta-state amplitude (Supplementary Fig. 4C) was consistent across the whole post early target interval, showing no obvious modulation with experimental condition and no significant difference for early vs. late targets. In contrast to the anticipatory interval, beta-state lifetimes (Supplementary Fig. 4D) now did modulate significantly in the same direction as power and fractional occupancy, with lifetimes being shorter for early compared to late targets directly following the target and being longer during the early target beta rebound phase (control group - first cluster: two-sided cluster p = .002; control group - second cluster: p = .004; PD group - first cluster: two-sided cluster p = .002, PD group - second cluster: two-sided cluster p = .038) but there were no significant differences between both groups.

The beta-state interval time (Supplementary Fig. 4E) again was modulated significantly by our experimental conditions, with the interval time being longer for early compared to late targets directly following the target (i.e. during a phase of lower beta power) and being shorter during the early target beta rebound phase (i.e. a phase of higher beta power; control group - first cluster: two-sided cluster p = .01; control group - second cluster: two-sided cluster p = .002; PD group - first cluster: two-sided cluster p = .01; PD group - second cluster: two-sided cluster p = .002). There were no significant differences in the early vs. late target contrast between both groups.

**Supplementary Analysis 5**

To investigate the influence of PD symptoms on each of our MEG and behavioural measures we performed correlational analyses with UPDRS-III score. For behaviour, we calculated Pearson correlation coefficients between UPDRS score and both the mean RT score (across all conditions) and the relative temporal validity effect at the early target. This analysis showed no significant correlations (Mean RT: r = 0.23, p = .37; Temporal Validity Effect: r = 0.14, p = .59).

For the MEG data, we calculated correlation coefficients between UPDRS score and the expect early vs. expect late difference in the last 200 ms of the cue – early target interval for each of our five MEG measures. As for behaviour, no significant correlations were found between UPDRS score and beta power (r = 0.149, p = .557) and between UPDRS score and our HMM-derived variables (fractional occupancy: r = - 0.034, p = .894; beta-state amplitude: r = -0.202; p = .421; beta-state lifetime: r = 0.392, p = .107; beta-state interval time: r = - 0.132, p = .600).
